# Learned Spatial Schemas and Prospective Hippocampal Activity Support Navigation After One-Shot Learning

**DOI:** 10.3389/fnhum.2018.00486

**Published:** 2018-12-04

**Authors:** Marlieke T. R. van Kesteren, Thackery I. Brown, Anthony D. Wagner

**Affiliations:** ^1^Educational Neuroscience, Institute of Brain and Behaviour Amsterdam, Vrije Universiteit Amsterdam, Amsterdam, Netherlands; ^2^Department of Psychology, Stanford University, Santa Clara, CA, United States; ^3^School of Psychology, Georgia Institute of Technology, Atlanta, GA, United States

**Keywords:** memory, schema, prior knowledge, spatial learning, hippocampus, medial temporal lobe, navigation, prospective planning

## Abstract

Prior knowledge structures (or schemas) confer multiple behavioral benefits. First, when we encounter information that fits with prior knowledge structures, this information is generally better learned and remembered. Second, prior knowledge can support prospective planning. In humans, memory enhancements related to prior knowledge have been suggested to be supported, in part, by computations in prefrontal and medial temporal lobe (MTL) cortex. Moreover, animal studies further implicate a role for the hippocampus in schema-based facilitation and in the emergence of prospective planning signals following new learning. To date, convergence across the schema-enhanced learning and memory literature may be constrained by the predominant use of hippocampally dependent spatial navigation paradigms in rodents, and non-spatial list-based learning paradigms in humans. Here, we targeted this missing link by examining the effects of prior knowledge on human navigational learning in a hippocampally dependent virtual navigation paradigm that closely relates to foundational studies in rodents. Outside the scanner, participants overlearned Old Paired Associates (OPA— item-location associations) in multiple spatial environments, and they subsequently learned New Paired Associates (NPA—new item-location associations) in the environments while undergoing fMRI. We hypothesized that greater OPA knowledge precision would positively affect NPA learning, and that the hippocampus would be instrumental in translating this new learning into prospective planning of navigational paths to NPA locations. Behavioral results revealed that OPA knowledge predicted one-shot learning of NPA locations, and neural results indicated that one-shot learning was predicted by the rapid emergence of performance-predictive prospective planning signals in hippocampus. Prospective memory relationships were not significant in parahippocampal cortex and were marginally dissociable from the primary hippocampal effect. Collectively, these results extend understanding of how schemas impact learning and performance, showing that the precision of prior spatial knowledge is important for future learning in humans, and that the hippocampus is involved in translating this knowledge into new goal-directed behaviors.

## Introduction

Prior knowledge strongly affects new learning (Bartlett, 1932; van Kesteren et al., 2012). New information that is stored in relation to prior knowledge structures (or schemas) is generally better remembered (McVee et al., 2005). The updating of knowledge networks is suggested to be mediated, in part, by retrieval of associative knowledge during learning (Preston and Eichenbaum, 2013), through an interplay between the medial temporal lobe (MTL) and medial prefrontal cortex (mPFC; van Kesteren et al., 2012; Gilboa and Marlatte, 2017). In rodents, where memory tasks are frequently spatial in nature, the facilitative effects of prior knowledge have often been attributed to computations within the hippocampus (Burgess et al., 2002; Hartley et al., 2003; Squire et al., 2004; Eichenbaum et al., 2007) in concert with representations in the mPFC (Tse et al., 2007, 2011; McKenzie et al., 2013, 2014; Richards et al., 2014). By contrast, human neuroimaging studies, which use predominantly non-spatial paradigms (though see van Buuren et al., 2014; Liu et al., 2017; Sommer, 2017), have often failed to observe hippocampal activity during the encoding of new schema-related knowledge (van Kesteren et al., 2010b, 2013; Brod et al., 2015). To bring the animal and human literatures closer together, it is of interest to examine the effect of existing spatial knowledge (Burgess et al., 2002; Hartley et al., 2003; McNamara et al., 2003) on new spatial learning in humans, and how this relates specifically to neural processing in the hippocampus and surrounding MTL cortices.

In the human spatial navigation literature, the role of the hippocampus in spatial processing has often been juxtaposed with functions attributed to other memory systems in the brain (Hartley et al., 2003; Iaria et al., 2003; Voermans et al., 2004; Doeller et al., 2008; Brown and Stern, 2014) and, of particular relevance to the present study, adjoining MTL cortex (Weniger and Irle, 2006; Ekstrom and Bookheimer, 2007; Weniger et al., 2010; Howard et al., 2011, 2014; Ekstrom et al., 2014). Across studies, the updating of spatial knowledge of an environment has been alternately associated with the hippocampus or parahippocampal cortex (Wolbers and Büchel, 2005; Weniger et al., 2010), with the divergence putatively being due to differences in allocentric vs. egocentric reference frames. Converging with foundational work on cortical declarative memory consolidation (McClelland et al., 1995), functional and neuropsychological data from studies of spatial navigation and remote spatial memory (Stefanacci et al., 2000; Rosenbaum et al., 2004; Moscovitch et al., 2006) suggest that long-term storage of learned spatial knowledge may rely on posterior parahippocampal cortex and a network of connected cortical regions, rather than the hippocampus. However, current perspectives from functional studies emphasize that, especially for new or recently formed memories, it is difficult to identify clean or natural dissociations between: (a) the navigational functions of the hippocampus and parahippocampal cortex; and (b) allocentric and egocentric reference frames (Ekstrom et al., 2014; Wolbers and Wiener, 2014). Reciprocal processing in the hippocampal-MTL cortex circuitry can give rise to both memory for navigational routes as “episodes” and spatial map knowledge which may ultimately and more gradually become “semanticized,” and it has been proposed that the mechanisms that give rise to these two forms of spatial memory may overlap with those underlying episodic memory and semantic knowledge in non-navigation settings (Buzsáki, 2005; Buzsáki and Moser, 2013). Consistent with these views, it may be the case that the combined, rather than selective, functions of the hippocampus and parahippocampal cortex may support recently learned spatial environment knowledge and enable new navigational experiences to update that knowledge.

Critically, both the hippocampus and parahippocampal cortex represent spatial goals from knowledge of overlearned virtual environments (Brown et al., 2016). Activity in both regions during navigational decision-making is also sensitive to the introduction of new routes/goal locations in a familiar environment (Brown and Stern, 2014; Brown et al., 2014). Such findings, along with evidence that new learning updates spatial goal representations in the rodent hippocampus (McKenzie et al., 2014), suggest that, although findings to date in humans mainly implicate extrahippocampal regions in the interaction between existing knowledge structures and new learning, the hippocampus and parahippocampal cortex may together be important for updating and accessing spatial knowledge structures in service of goal-directed behavior. As such, an important question is whether and how hippocampal and parahippocampal-dependent spatial retrieval mechanisms relate to prospective planning and goal-directed navigation for newly learned information that can be integrated into existing spatial knowledge structures.

Here, we targeted this question in humans, using a spatial navigation paradigm and fMRI to test whether: (1) as in prior rodent studies, existing spatial knowledge benefits new learning; and (2) whether the hippocampus and parahippocampal cortex, known to mediate spatial memory, support prospective planning of navigation based on new memories that relate to existing spatial knowledge. We designed a virtual navigation experiment, conceptually inspired by the event arena used in rodent research (Tse et al., 2007), to test whether the precision of prior spatial knowledge Old Paired Associate (OPA) predicts new, one-shot learning of a New object-location Paired Associate (NPA). Furthermore, we sought to examine the relationship between prospective hippocampal retrieval effects and one-shot NPA-learning facilitated by OPA knowledge. In the experiment, participants first learned the locations of faces (OPAs) through free navigational exploration of multiple, similar environments. After extensive OPA learning, they learned a new location (NPA) in each of the environments while undergoing fMRI. We hypothesized that greater OPA-knowledge precision would predict one-shot NPA-learning. Furthermore, we expected this behavioral effect to be facilitated by prospective retrieval effects in the hippocampus and parahippocampal cortex during navigational planning, supporting a role for the MTL system in incorporating rapidly integrated spatial experiences into planning and spatial goal localization.

## Materials and Methods

### Participants

Twenty-two neurologically healthy, right-handed participants with normal color perception between 18 years and 35 years old were recruited through Stanford’s University’s Sona-systems for subject recruitment, through flyers and through posting to a postdoctoral email list. Four participants did not complete the experiment due to virtual reality-induced motion sickness on the first day (Day 1), and two additional participants did not perform well enough to progress to the second day (Day 2)/scanning part of the experiment (see below for details). Accordingly, 16 participants (eight males; mean age 23.13 years, SD 4.60 years, range 18–35; 10 Caucasian, 2 American Indian/Native Alaskan, 2 Asian, 1 African American and 1 Hispanic) were scanned and entered in the analyses. Participants self-reported to have started to learn English on average at age 2.75 (SD 3.99) years and had 17.44 (SD 3.63) years of education. On average, participants self-reported to have slept 7.13 (SD 1.20) h between Day 1 and Day 2. This study was carried out in accordance with procedures approved by the institutional review board at Stanford University. All subjects gave written informed consent in accordance with the Declaration of Helsinki on both days and received monetary compensation for their time (maximum $90).

### Procedure

#### OPA Training (Behavioral)

**Figure 1:**
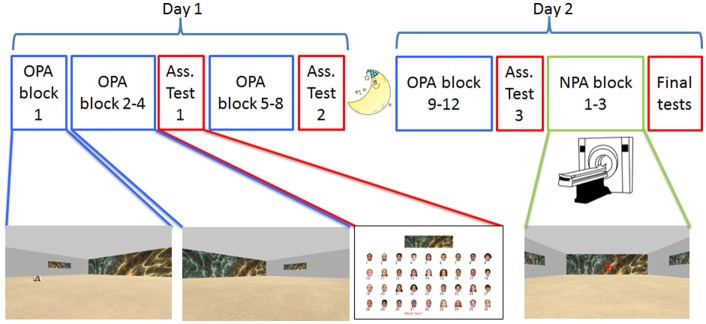
Experimental design. Participants were tested on two consecutive days, approximately 24 h apart. The paradigm was a 2D navigational paradigm in which participants were instructed to learn face-cue locations (Old Paired Associates, OPA; in blue) in 36 rooms with differential wallpapers. On Day 1 and the beginning of Day 2, participants learned OPA-locations on a laptop computer. During OPA block 1 they saw the OPA-location and just had to move to it, but for OPA blocks 2–12 they had to search for the hidden OPA-location. After OPA blocks 4, 8 and 12, participants received an associative memory task in which they were asked to pair the right face with the correct environmental/room wallpaper (in red). After finishing OPA training, participants underwent fMRI while they learned a new (New Paired Associates, NPA; in green) location for each room, this time without a face.

Participants were instructed to learn the spatial locations of 36 unique faces embedded in 36 unique rooms (see Figure 1) using a 2D virtual-reality navigation approach (Vizard VR, WorldViz). Rooms were square, sized at 40 (w) × 40 (l) × 10 (h) arbitrary units (a.u.); faces appeared on small, 3 × 3 × 3 a.u. cubes. The rooms only differed with respect to the wallpaper that was printed on one of the walls in full (40 × 10 a.u.) and on the other three walls as a smaller painting (12 × 3 a.u.). Wallpapers consisted of distinctive colored fractal patterns (collected from the internet); faces were colorized images of distinct Caucasian individuals (18 males, 18 females), and appeared on all four sides of a cube positioned at a pseudo-random location in each environment (see below under “Stimuli” section for specifics). On Day 1, participants learned the 36 room-face-location associations (Old Paired-Associates; OPA) across eight self-paced training blocks (OPA blocks 1–8); on Day 2, participants performed “top-off” learning across another four self-paced training blocks (OPA blocks 9–12).

To illustrate the procedure and learn how to navigate in the virtual rooms, participants first received practice on Day 1, which consisted of three trials in rooms with gray walls and white boxes at fixed positions. They used the “up,” “left” and “right” arrow buttons on the keyboard to navigate; they were not allowed to back up. Across the training trials on Days 1 and 2, participants were instructed to come up with their own strategy to learn the associations and to incorporate all three components (room/wallpaper, face and location) in memory. Furthermore, they were instructed to take the most direct route to the face when they knew its location, which allowed us to compare their traversed path length to the optimal path length Euclidian Distance (ED) from the starting position to the face’s location.

Within each training block, room order was randomized. At the beginning of each training trial within a training block, participants were cued with one of the 36 faces that was associated with that specific room (randomly assigned for each participant); the face was presented on a gray background for 1 s. They were then positioned in one of the corners of the corresponding room, oriented towards the room’s center. Participants were instructed to find the face, which was printed on the sides of a white cube positioned at a specific location in the room. In training OPA block 1, the face/cube was made visible throughout and participants only had to move to it to continue to the next trial. We adopted this design because it was discovered through behavioral piloting that participants would otherwise struggle to learn the 36 OPA locations in a reasonable training time. Although this renders block 1 of OPA and NPA learning (described below) incomparable, this effectively serves to boost OPA block 2+ beyond what was observed without this manipulation during piloting. In all subsequent training blocks (i.e., OPA blocks 2–12), the face/cube was hidden from sight and only appeared when participants arrived at its location (“arrival” was coded as appearing within a circle of 7 a.u. diameter). This meant that during the last 11 training blocks (OPA blocks 2–12), participants could find the face through memory or by exploring the room. A trial ended after the face was found and the participant walked into it (within a circle of <2 a.u.), after which the viewpoint rotated to the floor and was held in place with the ground texture and face stimulus centered in their field of view for 2 s, accompanied by the text “You found it, well done!.” After each training block of 36 trials, participants were allowed to take a short break; participants initiated the start of the next training block by making a button press.

Across each set of four Day 1 training trials for a room, all four starting positions were used once (order determined randomly without replacement) to discourage a strictly egocentric spatial learning strategy. For each room, the associated face, path length, optimal path length and search time were logged, as well as the total trajectory in x- and y-coordinates. After OPA block 4 and after OPA block 8, participants received an association test that directly probed their memory for the wallpaper-face associations (see “Memory Tests” section below for more details). These tests were designed to further encourage participants to form room/wallpaper-face-location associations (rather than simply face-location associations). The total procedure on Day 1 took on average about 2.5 h (range 2–3 h).

Day 1 performance was assessed by computing path efficiency (PE = traversed path length/optimal path length). Based on behavioral pilot experiments, we required participants to demonstrate strong knowledge of the faces’ locations at the end of Day 1. Specifically, the average PE across the final three Day 1 training blocks (i.e., OPA blocks 6–8) had to be <2 (using a weighted average of PE: OPA block 6*0.15, block 7*0.25, block 8*0.6); all but two participants reached this level of learning and were invited to come back for Day 2 ~24 h later (mean lag of 23.97, SD 0.39 h). These criteria were set *a priori* and were calculated to differentially weight the latest block, under the assumption that it provides the most up-to-date measure of learning, while not solely depending on performance in the latest blocks due to concerns about variability due to nuisance factors (e.g., possible effects of fatigue; transient fluctuations in motivation/attention; etc). On Day 2, participants performed the four “top-off” OPA training blocks (OPA blocks 9–12), which were identical to OPA blocks 2–8 in structure, but were randomized (such that the order of, e.g., block 2 on Day 1 was the order of block 3 on Day 2), starting again once from all four starting positions. Following training, participants performed another test of the wallpaper-face associations before they proceeded to the critical fMRI scanning session. Note that all Day 1 and the Day 2 training blocks and wallpaper-face associative memory tests were administered in a behavioral testing room, four floors above the MR scanning suite.

#### NPA Learning (fMRI)

On Day 2 and following OPA learning, participants underwent fMRI scanning while they learned a NPA for each of the 36 rooms. All general task variables related to navigating the rooms (i.e., the size of the rooms, the fractal wallpapers, navigation speed, et cetera) were held constant between the OPA training and NPA learning. During NPA learning, participants were instructed that they would be navigating all rooms again three times, but that the hidden faces were no longer in the rooms. Instead, there would be a new location at which a hidden white box was placed and they were instructed to find and learn the location of the box. Critically, this time the white box was not visible during the first NPA block, and thus they had to search for it from the start. Moreover, to equate visual input at the start of each trial, participants did not start at the corners of the room as during OPA training, but instead always started positioned against the middle of the wall opposite to the wall covered in full with wallpaper. At the beginning of each trial, participants were placed in the room facing the opposite wall and were informed that they had to wait 8 s before they could start searching for/navigating to the NPA; this 8-s period thus provided an opportunity for participants to plan their navigation (*planning period*). The planning period was further signaled by a red fixation cross in the middle of the screen that disappeared after 8 s; the response buttons were locked during this period, ensuring the participants did not move in the environments. At the end of each trial, corresponding to when the participant arrived at the NPA’s location, a white square was presented in the middle of the screen for 2 s (*goal-arrival period*). A 7-s inter-trial interval (ITI), consisting of a white fixation in the middle of the screen, separated each trial; following the ITI, the fixation cross turned green, indicating that a new trial was about to start; onset of the next trial was aligned with the onset of the next TR. For each room, in addition to the associated OPA face, path length to the NPA, optimal path length to the NPA and NPA search time, onset times were logged for both the planning period and the goal-arrival period.

Participants lay supine in the scanner and viewed the screen through a mirror on top of the coil. Head movement was minimized using padding around the head and, when participants did not object, masking tape was placed on the forehead and attached to the sides of the coil. This tape provided movement feedback to the participant. To navigate the environments, participants used a 4-key button box (fORP 932, Current Designs) under their right hand. During practice (three trials) in the scanner, a structural scan was run. As during OPA training, participants were shown the locations of the white boxes within novel rooms, enabling them to practice using the navigation buttons (the first three buttons of the button box: left (forefinger), forward (middle finger), right (ring finger)). Subsequently, each NPA block consisted of one trial in each of the 36 rooms; these trials were randomly divided into two sets of 18 trials, which corresponded to two separate scanning runs. Thus, participants performed six runs of 18 rooms each, allowing each room to be repeated three times. After each run, participants were given the possibility for a short break of a few minutes in the scanner. When all six runs were finished, participants completed two 6-min localizer tasks (see below for more details). Then they were taken out of the scanner and were given a short break. Finally, they were taken back to the behavioral testing room to take two more memory tests and fill out some questionnaires (see below under “Memory Tests” section). In total, participants spent between 3 h and 4 h on Day 2: ~1 h for OPA blocks 9–12, ~2 h for the critical NPA learning phase in the scanner, and ~0.5 h for the final memory tests and questionnaires.

#### Memory Tests

All memory tests were presented using PsychToolbox 3.0.10^1^ in Matlab (MathWorks) on a laptop. The wallpaper-face association tests that were interleaved throughout OPA training (i.e., after OPA blocks 4, 8 and 12) probed associative memory for the face cued with the wallpaper. On each test trial, a wallpaper cue was shown on top of the computer screen (the 36 wallpaper cues were presented in random order). Below each wallpaper cue, all 36 faces were shown in random order in four rows of nine faces (see Figure 1), all accompanied by a number (1–36). The participants were instructed to type the number of the associated face and press “Enter” to proceed to the next trial. Trials were self-paced and reaction time was logged.

Following scanning on Day 2 (see “Procedure” section above), participants completed two final memory tests, probing final spatial memory for the NPA and OPA given the wallpaper or the OPA (face cue) outside of virtual navigation. In the first test, participants were shown a birds-eye view of the room with the wallpaper cue printed above in random order. Participants were instructed to click on the room where the NPA location was located as related to the wallpaper. After clicking, they proceeded to the next room. In the second tests, participants were shown the same view of the room, but now with a gray wallpaper and the associated OPA face cue, again in random order. They were instructed to first click where the OPA was located, after which the OPA was moved to the right location. Then, the participants were instructed to click where the associated NPA location was within the same room. After these final memory tests, participants filled out questionnaires that probed: (a) general navigating strategies Questionnaire on Spatial Representation (QSR; Pazzaglia and De Beni, 2001); and (b) strategies specific to this paradigm. Participants were also asked to report the number of hours slept between Day 1 and Day 2.

#### Stimuli

All rooms had gray walls, a gray ceiling and a beige textured floor. Corners were accentuated with a black line to make them more visible. Wallpaper fractals and faces were selected based on behavioral piloting that ensured each stimulus was easily identifiable and distinguishable from the others. Wallpaper-face pairings for each environment were randomized for each participant.

For each participant, the OPA locations were pseudo-randomly assigned to rooms without replacement, taken from a set of 36 predetermined locations that were calculated given a few boundary conditions: (1) locations that were too close to the walls were excluded, making sure participants could walk around the box from all sides (3 a.u.); (2) a location needed to have at least a 3-s walking distance from any corner, which ensured that participants did not immediately run into it when searching/navigating; and (3) OPA locations were calculated to be approximately evenly distributed across the environments within the aforementioned constraints (such that, across environments and participants, the floor space was approximately evenly tiled with OPA locations). For NPA locations, we calculated new location coordinates using similar boundary conditions, but the locations were positioned to have at least a 3-s walking distance from the starting position used for NPA learning. Critically, each participant’s NPA locations were also constrained to be at least 7 a.u. from the same environment’s assigned OPA location and at least 2 a.u. from all other OPA locations used for that participant (to prevent across-environment OPA-NPA overlap). NPA locations were not the same, for a given environment, across participants.

#### Localizer

Following the NPA scanning blocks, participants performed a functional localizer task (consisting of two 360 s fMRI runs) to determine subject-specific face, scene (all outdoor scenes to maximize distinction with the indoor room in our main task), and room-cue related brain activity. Because the localizer scans were performed to support the testing of hypotheses that are not the focus of the present manuscript, we refrain from reporting the details of these scans as they are not germane.

#### MRI Parameters

Participants were scanned at the Stanford Center for Cognitive and Neurobiological Imaging (CNI) using a 3T GE Discovery MR750 scanner and a 32-channel head coil (Nova Medical). A T2*-weighted echo planar imaging sequence (TR = 2 s; TE = 30 ms; flip angle = 77 degrees; acquisition matrix = 80 × 80; 42 oblique slices oriented along the AC-PC axis; 2.9 × 2.9 × 2.9 mm spatial resolution) was used for both the experimental and localizer scans. The number of scan volumes differed across participants and across runs because of the variable path lengths taken to the NPAs. Additionally, a 3D T1-weighted anatomical scan was acquired for normalization and activity localization (TR = 7.24 ms; TE = 2.78 ms; flip angle = 12 degrees; acquisition matrix = 256 × 256; 186 sagittal slices; 0.9 × 0.9 × 0.9 mm spatial resolution).

#### fMRI Preprocessing

Raw fMRI data from the spatial navigation/NPA learning task were preprocessed using SPM12^2^. First, the functional data were slice time corrected to the middle slice. Second, motion correction was performed by using iterative rigid body realignment to minimize the residual sum of squares between the first and all other functional volumes. Third, rigid body co-registration to the corresponding individual T1 structural image was performed using mutual information optimization. Fourth, segmentation of the T1 structural image into gray matter, white matter and cerebrospinal fluid (CSF) was performed. Fifth, data were spatially normalized using DARTEL (Ashburner, 2007; Yassa and Stark, 2009), where a common template was calculated based on the average of all individual segmented T1 structural images (gray and white matter). Finally, data was spatially smoothed at 8 mm FWHM. To further control for the influence of artifacts, we utilized the Artifact Detection Tools (ART^3^) to identify signal intensity and combined motion-signal intensity outliers in conjunction with the movement parameters calculated in SPM. Artifacts and motion parameters were included in the single-subject first-level models (see below).

#### Behavioral Analyses

**Figure 2:**
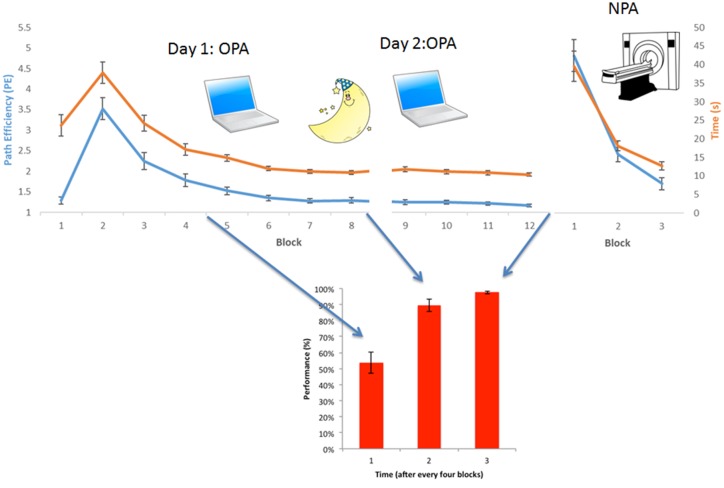
Behavioral results OPA-training and NPA-learning. Average group-level behavioral results. (Upper) Normalized path length (or Path efficiency, PE, in blue) and time needed to find the cue (in orange) for each block, both during OPA and NPA learning blocks. (Lower) Associative wallpaper-face memory was tested after every fourth OPA block. OPA, NPA and associative performance significantly improved over blocks, revealing robust learning. Importantly, NPA learning (NPA blocks 2 and 3) was significantly faster than OPA learning (OPA blocks 2 and 3), as supported by a significant condition × block interaction.

We first established that OPA and NPA learning rates (Figure 2) were significant (non-zero slopes) by averaging the normalized PEs of the 36 rooms for each block. A repeated-measures ANOVA, with 11 measurements for OPA training (the first block in which the location was visible was excluded) and three measurements for NPA learning, was used to examine learning (both PE and time to cue) of the OPA and NPA locations over blocks using IBM SPSS Statistics 24. Pearson correlations were used to examine the relationship between PE and time to get to cue.

**Figure 3:**
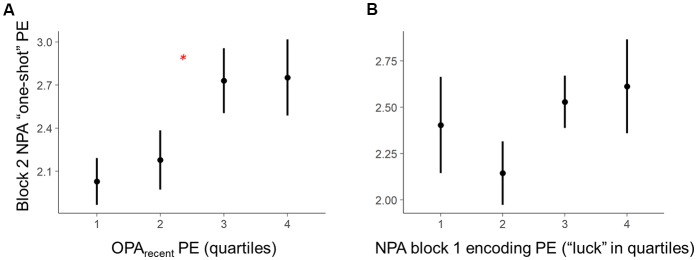
Within-subject trial-level behavioral predictors of one-shot NPA learning. Quartiles reflect within-subject binning of environment-by-environment measures into quartiles for visualization purposes only (Linear Mixed Effects, LME analyses used continuous performance differences across each environment). **(A)** Performance on the most recent retrieval-practice experience with OPA items (i.e., OPA block 12) predicted NPA PE after one-shot learning (i.e., NPA block 2 performance). **(B)** There was a complex, curvilinear relationship between “luck” in NPA location search in NPA block 1 and one-shot learning success (controlled for in our statistical analyses). *p < 0.05.

To test for a “schema” learning benefit of OPA knowledge on subsequent NPA learning, two analyses were conducted:

1. First, as an initial coarse test of the hypothesis, a repeated-measures ANOVA examined whether performance on NPA blocks 2–3 was significantly greater than on OPA blocks 2–3. While superior NPA vs. OPA performance could reflect the benefits of prior knowledge of the OPA’s location within a room during NPA learning, other accounts are also viable (e.g., learning to learn within the task). A room-level test (analysis #2) is needed to directly examine the hypothesis.
2. Second, we conducted a room-level test, analyzing whether room-by-room OPA performance during training predicted one-shot encoding success for a new spatial association (i.e., NPA learning) in the same environments (Figure 3A). Given that memory retrieval has been linked with mnemonic malleability (Schlichting and Preston, 2015; van Kesteren et al., 2016; Lee et al., 2017), we first examined the relationship between one-shot NPA learning and prior knowledge as a function of the precision (PE) of the most recent OPA retrieval experience in an environment (OPA block 12; hereafter “OPA_recent_”). Second, we explored in a separate model how trial-invariant spatial memory performance for each environment, weighted over the last four blocks (rather than the most recent experience; within-room average PE for OPA blocks 9–12 — “OPA_average_”; see below for details), relates to one-shot NPA learning.

The second set of analyses leveraged Linear Mixed Effects (LME) modeling in R Core Team (2012) to test the predicted behavioral relationships as fixed effects, while accounting for random effects and mediating factors. One powerful aspect of LME for group-level inference of this type is that we can better characterize predicted fixed effects (e.g., group-level OPA-NPA performance relationships) by accounting for a range of random effects—specifically, not only random intercepts for participants, but random slopes for participants in the tested relationship, as well as “item effects” (random, undesired systematic effects of room). When these effects are not accounted for, such random effects can color interpretation of the fixed effects. Specifically, examination of the data revealed a complex relationship between “luck” when searching the environments during the first block of NPA learning (i.e., NPA block 1) and NPA performance during the second block (i.e., NPA block 2; see Figure 3B). Because such instances of “luck” have dramatic biasing effects on our measure of NPA one-shot learning (i.e., NPA block 2 PE), they complicate a test of the relationship between OPA memory and NPA learning. Accordingly, we divided NPA block 1 PE data into two distinct components and included them as additional predictors in our LME models: (1) “lucky shots,” defined as trajectories within 15% of the optimal path length, at which point paths began to approximate direct routes to the goal. This cutoff was derived from an initial computation of Frechet distance, as implemented in the Similarity Measures R package (Alt and Godau, 1995), which provides a measure of how far a trajectory (participant’s true path) deviates from a reference trajectory (optimal path). In the context of our open field experiment, where trajectories can vary considerably in their shape, this is an ideal measure for characterizing when a true path was a spatially close match to the optimal trajectory. A Frechet Lucky Shot cutoff of 3.5 units was set based on the radius in which the hidden goal locations would become visible, which effectively meant that the participant searched for the NPA on a path that deviated from the optimal direct trajectory so little that they could not miss the target. In the standard PE metric used in the literature, this cutoff equated to ≤ 15% PE (with one exception, a case in which a participant accrued 31% PE by zigzagging across the optimal path. Using this participant’s PE as cutoff, however, would be quite liberal, resulting in many indirect paths being classified as lucky shots); and (2) residual block 1 PEs. These block 1 PE residual data exhibited a linear relationship with NPA block 2 performance.

Respectively, “lucky shots” composed 12.0% of trials and were associated with worse NPA one-shot learning and longer block 1 residual PEs were associated with worse one-shot learning (evident in the overall one-shot learning*encoding PE relationship in Figure 3B). Although Lucky Shots were defined by a PE threshold, it is worth noting that they did not exhibit any relationship to the proximity of the hidden NPA location (Lucky Shot optimal path length: 12.0–36.5 [mean 20.7 a.u.] vs. 12.1–37.6 [mean 24.2 a.u.]). This may be because participants tended to employ distinctive search strategies in NPA block 1 (e.g., spiraling) that could give rise to a lucky, fairly direct trajectory towards the target. Controlling for these two dimensions of NPA block 1 variability when examining the relationships between OPA_recent_ and OPA_average_ and NPA one-shot learning allowed us to more directly test whether OPA memory (i.e., prior knowledge of a relevant location within a room) predicts successful one-shot NPA learning (i.e., learning the newly relevant location within a room). The OPA_recent_ and OPA_average_ LME analyses both treated participant and environment/room (i.e., item effects) as random intercepts, and models were estimated using a restricted maximum likelihood (REML) approach. Maximal random effects (intercepts and slopes) allowed by the data were included in each model (Barr et al., 2013); if the maximal model could not be fit due to a lack of observations, or would not converge (after changing optimizers and increasing the number of iterations), the model was pruned by iteratively removing random slopes (starting with interaction terms). The package lmerTest (version 2.0-33) was used for estimating *p*-values with Satterthwaite approximations for degrees of freedom for one-sample *t*-tests from mixed effects models.

In the OPA_recent_ model, the OPA_recent_ predictor of NPA learning reflected PE for each room on the final trial of OPA training (i.e., OPA block 12) on Day 2, prior to NPA learning in the scanner. As noted above, we also considered the possibility that NPA learning may differentially relate to OPA_recent_ and a *trial-invariant* index of OPA spatial knowledge; in contrast to OPA_recent_, an average estimate of trial-invariant knowledge on Day 2 may better reflect the amount of stable, consolidated spatial knowledge for each environment prior to engaging in NPA learning (i.e., the learned “schema”). This distinction was theoretically significant to us because the schema learning literature posits that the hippocampus is less important for retrieval of learned schemas, being instead more important for event-related learning (van Kesteren et al., 2012). Although we cannot quantify consolidation in the present data, our OPA_average_ measure emphasizes trial-invariant (stable) OPA performance after a night’s consolidation. The prior schema learning literature would therefore predict OPA_average_ performance would reduce hippocampal dependence for retrieval of even newly-integrated knowledge of the environment—which our data provide evidence for (see “Results” section below). By contrast, OPA_recent_ encompasses this knowledge but also reflects vagaries of recent episodic experience (e.g., fatigue; cross-environment mnemonic interference; continued learning; and relearning driven by preceding errors that occurs on Day 2 leading up to this event) that could contribute to mnemonic malleability associated with this retrieval attempt (Schlichting and Preston, 2015; van Kesteren et al., 2016; Lee et al., 2017) and thus further mediate NPA learning.

This OPA_average_ predictor was defined as the weighted mean of the four OPA training trials for each room on Day 2 (i.e., OPA blocks 9–12), prior to NPA learning in the scanner (linear weighting towards end of practice). We reasoned that the weighted mean should downweight initial trials, because on those trials participants had to reorient to the environments learned the day before and they were more prone to errors. Thus, we weighted away from the “refresher” state in the beginning and towards the maximally learned schema (we note, however, that the effect of weighting the average was ultimately minimal, with the correlations within-subjects between weighted and non-weighted room-by-room metrics being ~0.96). We removed single outlier events (if any) from each room’s mean, using Dixon’s Q to identify spikes in PE with a 95% confidence interval (note: with four trials per room, two or more spikes in performance could not be considered outliers). Although the OPA_recent_ and OPA_average_ measures are inherently correlated, and although participants demonstrated strong OPA knowledge through high performance on these Day 2 training blocks, on average over 35% of the within-subject OPA_recent_ variance across rooms was not explained by OPA_average_ performance. This substantial variability in performance across rooms on the final OPA training trial could, in theory, influence subsequent NPA learning above and beyond trial-invariant OPA_average_ knowledge on Day 2, and our data (below) suggest this is the case.

#### fMRI Analyses

Functional data from the third and fourth NPA runs (which correspond to NPA block 2) were combined into one model that modeled each trial onset (planning period) as separate regressors using a delta function. The present study’s functional analyses (detailed below) focus on data from Block 2, because (1) prospective retrieval signals for NPA locations would not exist in Block 1; and (2) the relationship between Block 1 activity and subsequent performance cannot be clearly interpreted due to the fact that subsequent navigation in Block 1 was characterized by wandering behavior in search of the unlearned goal location. In our GLM, we also added a regressor for the goal-arrival in each room, modeled as a 2-s boxcar function and the navigation onset time, modeled with a delta function. Nuisance regressors included: the movement and artifact regressors, a regressor modeling scan run, and a regressor to account for global variance.

In order to directly address our question of whether and how prospective memory signals manifest in hippocampus and parahippocampal cortex as a function of schema-related learning, we extracted single-trial parameter estimates derived from our first-level models from bilateral hippocampal and parahippocampal cortex regions of interest (ROIs) for the NPA block 2 planning period. Additionally, given evidence that posterior hippocampus [and the corresponding putative “posterior medial” system (Ranganath and Ritchey, 2012)] may be preferentially recruited during episodic retrieval of detailed scene/relational information (Ranganath and Ritchey, 2012; Poppenk et al., 2013), we further segmented the hippocampus into head, body and tail regions. Anatomical ROIs were manually traced in MNI space on the group averaged DARTEL template-normalized brain using the ITK-SNAP software package^4^ (Yushkevich et al., 2006) using established procedures (Insausti et al., 1998; Pruessner et al., 2000, 2002; Duvernoy, 2005; Preston et al., 2010; Brown et al., 2014). Given recent interest in the interplay between the MTL and mPFC in updating of knowledge networks (van Kesteren et al., 2012; Gilboa and Marlatte, 2017), we also conducted an exploratory analysis of mPFC recruitment during NPA block 2 planning. Due to variability in functional loci across prior studies, we defined a bilateral mPFC ROI that encompasses prior observations in the literature (van Kesteren et al., 2012) by implementing the more ventral of two medial prefrontal nodes (in yellow; 17-network) associated with the default mode network, explicitly masked to the medial wall of the PFC (Yeo et al., 2011).

The single-trial parameter estimates were entered into separate LME models for each MTL ROI (hippocampal head, body and tail, and parahippocampal cortex), to predict NPA performance in the second block (i.e., a measure of one-shot learning; see above). We employed LME modeling to render our statistical approach involving fMRI data comparable to the primary behavioral “schema learning benefit” analysis using LME. This enabled our fMRI analyses to examine activity as a key predictor while controlling for other factors (e.g., hippocampal activity predicting one-shot NPA learning while accounting for lucky shots and NPA block 1 naïve search performance). In these analyses, parameter estimates replaced OPA_recent_ and OPA_average_ measures in the LME models described above, to test for the predicted relationships between prospective (planning period) MTL activity and subsequent performance after one-shot learning, holding “luck” during the one-shot learning event constant. We also entered both OPA performance and parameter estimates into omnibus models, aimed at testing whether activity and OPA performance independently predict NPA performance when the other metric is accounted for.

Lastly, because we observed that OPA performance and prospective hippocampal activity both predict NPA performance after one-shot learning (see below), it was of interest to examine the relationship between OPA performance and prospective hippocampal activity during NPA block 2. To address this question, we modified the two LME models used to predict NPA performance from OPA (holding NPA luck constant) to test whether OPA performance predicts prospective MTL activity after one-shot learning.

## Results

### Behavioral Results

Repeated-measures analysis revealed that both OPA learning (blocks 2–12 PE; Greenhouse-Geisser: *F*_(1.54, 23.01)_ = 54.88, *p* < 0.001) and NPA learning (blocks 1–3 PE) were significant over blocks (Greenhouse-Geisser: *F*_(1.16, 17.39)_ = 63.51, *p* < 0.001). Also time taken to find the OPA (Greenhouse-Geisser *F*_(1.91, 28.63)_ = 71.33, *p* < 0.001) and NPA (Greenhouse-Geisser *F*_(1.05, 15.75)_ = 43.23, *p* < 0.001) locations decreased significantly. PE and time correlated strongly (OPA *r*_(14)_ = 0.91, *p* < 0.001; NPA *r*_(14)_ = 0.99, *p* < 0.001). Associative memory for the wallpaper-face associations also significantly increased across OPA training: (Greenhouse-Geisser: *F*_(1.46, 21.88)_ = 42.74, *p* < 0.001). Importantly, NPA learning (indexed by block 2 and 3 PE) was significantly faster than OPA learning (main effect of condition (OPA vs. NPA): *F*_(1, 15)_ = 17.40, *p* = 0.001; see Figure 2). There was a significant interaction between condition and block (*F*_(1, 15)_ = 7.39, *p* = 0.02), indicating that the difference between OPA and NPA performance became smaller as NPA performance approached ceiling (see Supplementary Table S1 for full behavioral summary split by gender).

The finding of superior NPA vs. OPA learning is noteworthy because OPA training benefitted from the OPA object/location being visible in the initial encoding block (i.e., OPA block 1); by contrast, the new goal location was not visible during NPA block 1. This design difference meant that: (a) both OPA and NPA block 2 involved an attempt to retrieve a location participant had navigated to in the immediately preceding block (the presence/precision of a one-shot memory trace); but (b) OPA block 2 benefited from participants having been able to encode the OPA location in block 1 from the trial outset relative to any reference point in the environment. Despite this, NPA learning was substantially accelerated after the same number of repetitions. That said, it is worth noting that this benefit of prior OPA training on NPA learning may be accounted for, at least in part, by: (a) a general learning-to learn benefit; and (b) the fact that NPA trials began from the same start position across rooms and across blocks. Without knowing the relative impact of these respective benefits to OPA and NPA learning, this initial coarse comparison of learning rates should be interpreted with some caution. A room-level test is needed to control for these alternatives and directly examine the hypothesis that prior knowledge benefits new learning in a continuous manner, and analyses based on this approach (reported below) offer our primary evidence for schema-benefitted learning.

### Post-scan Memory Tests

Average performance measures for the post-scan tests were evaluated by calculating the average ED from the correct location. In the test where NPA-location memory was probed by cueing the wallpaper, mean ED was 7.48 (SD 2.38). When cueing with the OPA face, performance was significantly poorer (mean ED 8.55 (SD 3.28); *t*_(15)_ = −3.11, *p* < 0.01). OPA performance was better than both these values [mean ED 6.32 (SD 1.79)] and correlated positively with both (vs. NPA-location cued memory *t*_(15)_ = 2.58, *p* < 0.05, *r*_(14)_ = 0.66, *p* < 0.01; vs. OPA-face cued memory *t*_(15)_ = 3.96, *p* = 0.001, *r*_(14)_ = 0.76, *p* = 0.001). OPA memory thus did not interfere with NPA learning.

Critically, holding “luck” during initial NPA object search constant [Figure 3B, note attenuated NPA learning in relation to NPA block 1 quartile 1 as well as extended search events (quartile 3–4)], analyses revealed that the precision of knowledge of the previously learned goal locations (OPA_recent_) predicted the degree of success of one-shot learning of new goal locations (*t*_(21.27)_ = 2.36, *p* = 0.03; Figure 3A). By contrast, in the model using OPA_average_ instead of OPA_recent_, OPA_average_ did not significantly predict one-shot NPA learning (*t*_(13.83)_ = 1.69, *p* = 0.11), suggesting one-shot NPA learning success is more strongly tied to the mnemonic and cognitive state of the most recent OPA retrieval experience.

### fMRI Results

**Figure 4:**
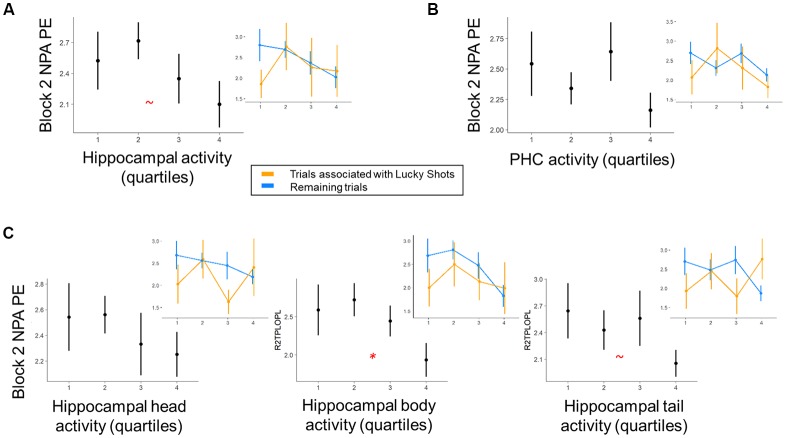
Within-subject trial-level relationship between region of interest (ROI) activity and NPA performance after one-shot learning. Quartiles reflect within-subject binning of environment-by-environment measures into quartiles for visualization purposes only (Linear Mixed Effects, LME analyses used continuous performance differences across each environment). **(A)** During NPA block 2 prospective planning (i.e., after one-shot learning), there was a marginal relationship between hippocampal activity and navigation performance. **(B)** This relationship was non-significant in parahippocampal cortex. **(C)** Within hippocampal subdivisions, prospective planning activity in the hippocampal body significantly predicted NPA block 2 navigation performance. Inset graphs: paralleling exploration of our behavioral data, “Lucky Shots” (orange; controlled for in our analyses) altered the relationship between activity and one-shot NPA performance. *p < 0.05, ^~^*p* < 0.1.

We next investigated the relationship between MTL activity during NPA planning after one-shot learning (i.e., planning period during NPA block 2) and subsequent NPA memory performance (Figure 4). After one-shot NPA learning, prospective planning activity across the hippocampus marginally predicted subsequent NPA spatial memory accuracy (*t*_(12.52)_ = 2.01, *p* = 0.07; Figure 4A). Within hippocampal subdivisions, activity in the hippocampal body significantly predicted NPA performance (*t*_(17.59)_ = 2.41, *p* = 0.03); this relationship was marginal in the hippocampal tail (*t*_(9.42)_ = 2.03, *p* = 0.07), and nonsignificant in the hippocampal head (*t*_(10.30)_ = 1.26, *p* = 0.24; Figure 4C). It is important to acknowledge, however, that this finding in the hippocampal body would not survive correction for multiple comparisons at traditional significance thresholds (*p* < 0.05), and therefore, despite *a priori* motivation for examining the hippocampus on the basis of rostro-caudal subdivisions, interpretative caution is warranted. Whereas hippocampal activity, particularly in the hippocampal body, exhibited prospective performance-related signals after one-shot NPA learning, the relationship between activity and subsequent performance in adjacent parahippocampal cortex was nonsignificant (*t*_(14.87)_ = 0.88, *p* = 0.39; (Figure 4B). Indeed, despite hippocampal and parahippocampal activity exhibiting the same qualitative relationship with NPA performance, when we included parahippocampal activity as a predictor in the same model, hippocampal body activity maintained a marginally significant relationship with subsequent NPA performance (*t*_(18.70)_ = 1.90, *p* = 0.07). Likewise, the hippocampal body remained a significant predictor of subsequent NPA performance, when controlling for activity in the hippocampal head (*t*_(51.61)_ = 2.37, *p* = 0.02), although this was not the case when hippocampal tail activity was held constant (*t*_(25.10)_ = 1.64, *p* = 0.11). Consistent with our examination of the tail and head as individual predictors, when the tail and head were included in the same model, neither were significant predictors of NPA performance (*p*’s = 0.27 and 0.95, respectively). Note that we were restricted to examining interactions between our different ROIs in this pairwise manner because the models failed to converge when made more complex. In our exploratory analysis of mPFC activity, mPFC activity was significantly positively correlated (functionally coupled) with the MTL ROIs (all *p*s < 0.001). However, mPFC activity did not significantly predict performance on the upcoming trial after one-shot learning (*t*_(29.66)_ = 1.68, *p* = 0.10).

**Figure 5:**
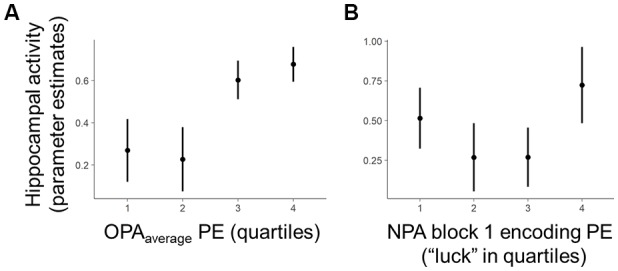
Within-subject relationship between OPA performance and hippocampal activity. Quartiles reflect within-subject binning of environment-by-environment measures into quartiles for visualization purposes only (LME analyses used continuous performance differences across each environment). **(A)** Within the hippocampus, there was a negative relationship between planning activity and OPA_average_ performance that significantly interacted with “luck” during initial NPA encoding. **(B)** Controlling for the effect of Lucky Shots, when participants had better OPA_average_ performance and encountered the NPA location quickly during NPA block 1, they recruited the hippocampus and its subdivisions less on NPA block 2 when planning navigation to the NPA location.

Given that OPA performance and, to a more modest degree, prospective hippocampal activity after one-shot learning predicted NPA performance after one-shot learning, it was of interest to examine the relationship between OPA performance and hippocampal activity (Figure 5). Interestingly, there was no evidence across MTL ROIs for a relationship with OPA_recent_ performance (*p*s > 0.70). However, within the hippocampus there was a negative relationship between activity and OPA_average_ performance (Figure 5A) that significantly interacted with search efficiency during NPA encoding (Figure 5B). Specifically, when participants had better trial-invariant OPA knowledge, if they encountered the NPA location more quickly during NPA search, they recruited the hippocampus significantly less when planning navigation to the NPA location on the subsequent trial (hippocampus: *t*_(568)_ = 2.63, *p* = 0.01; head *t*_(299.5)_ = 2.65, *p* = 0.01; body: *t*_(565.6)_ = 2.48, *p* = 0.01; tail: *t*_(568)_ = 2.39, *p* = 0.02; PHC: *t*_(567.9)_ = 1.55, *p* = 0.12; mPFC: *t*_(5)_ = 2.04, *p* = 0.10). The main effects for this relationship did not exceed trend levels (*p*s > 0.08). This finding is noteworthy because our data demonstrate that both hippocampal activity and better OPA_recent_ performance positively predicted improved NPA performance after one-shot learning.

Given the outcomes of the analysis relating OPA memory to prospective MTL activity, we hypothesized that hippocampal activity and OPA_recent_ performance may independently predict NPA performance when the other metric is accounted for. Due to the relatively large number of modeled parameters, the results of this exploratory follow-up analysis should be interpreted with caution, but did lend support to this idea. That is, when both MTL activity and OPA_recent_ performance were entered as predictors in the same model, their independent relationships with NPA performance demonstrated above remained significant or marginally-significant. OPA_recent_ and hippocampal activity both predicted NPA performance (hippocampus: *p* = 0.05, OPA_recent_: *p* = 0.05). OPA_recent_, but not hippocampal head activity, marginally predicted NPA performance (head: *p* = 0.28, OPA_recent_: 0.051). OPA_recent_ and hippocampal body activity significantly and marginally predicted NPA performance (body: *p* = 0.023, OPA_recent_: 0.052). OPA_recent_ and hippocampal tail marginally predicted NPA performance (tail: *p* = 0.08, OPA_recent_: *p* = 0.06). OPA_recent_ remained a significant predictor of NPA performance when PHC activity was held constant (PHC: 0.26, OPA_recent_: *p* = 0.05). In contrast, when OPA_average_ was instead entered in the model, neither MTL activity nor OPA_average_ knowledge were significant predictors [holding OPA_average_ constant, a marginal NPA performance relationship with activity was observed in the hippocampus and hippocampal body [*p* = 0.09 and *p* = 0.06]; all remaining *p*s for MTL activity and OPA_average_ > 0.18].

## Discussion

Our analyses produced several key findings: (1) a continuous measure of the precision of OPA prior spatial knowledge predicts one-shot NPA learning; (2) prospective hippocampal planning activity emerges after one-shot NPA learning that predicts the precision of memory-guided navigation; and (3) hippocampal engagement during NPA retrieval after one-shot learning is reduced as a function of an interaction between greater trial-invariant OPA prior knowledge and how quickly the NPA search during encoding was achieved. These findings extend the sparse literature on how prior spatial knowledge affects new navigational learning and performance in humans. We build on this important behavioral finding to establish a link between prospective memory signals in the hippocampus and initial one-shot learning of new knowledge that is facilitated by prior knowledge.

Our paradigm, while a simplified instantiation, was designed to conceptually parallel those used in rodent studies of the influences of prior knowledge on new learning (Tse et al., 2007, 2011). We demonstrated clear evidence for a continuous behavioral effect of prior spatial knowledge on new spatial learning in humans that parallels previous rodent (Tse et al., 2007; McKenzie et al., 2013, 2014; Richards et al., 2014) and human (van Kesteren et al., 2010a,b, 2013; van Buuren et al., 2014; Wagner et al., 2015; Sommer, 2017) studies which, respectively, show memory enhancement for newly learned information built on prior spatial and non-spatial knowledge. As such, the present behavioral findings not only provide a novel link between continuous measures of spatial knowledge precision and new spatial memory learning at a trial-by-trial, environment-by-environment level in humans, but they also help bridge findings from extant rodent and human research that has been predominantly conducted in different spatial and non-spatial domains.

The ability to flexibly plan for the future is critical for achieving goals, and prominent theories posit that one must access knowledge from prior experiences to construct detailed simulations when planning for the future (Addis et al., 2007; Buckner and Carroll, 2007; Schacter and Addis, 2009). Spatial navigation is a ubiquitous real-world example in which prior knowledge informs prospection and planning of future actions, and extant data in rodents and humans support a role for the hippocampus during prospection over spatial navigation (e.g., Johnson and Redish, 2007; Wikenheiser and Redish, 2015; Brown et al., 2016). An open question is how rapidly prospective navigational retrieval activity emerges across learning, and how these signals—critical for goal-directed behavior—relate to interactions between encoding experiences and prior knowledge. The present findings demonstrate that at the neural level, prospective hippocampal signals emerge after one-shot learning and predict subsequent navigation to new spatial goals. More specifically, greater activity in the hippocampal body during prospective navigational planning to the NPA location in block 2 (i.e., after one-shot NPA-learning) predicted more precise subsequent navigation to the NPA. By contrast, although prior literature implicates MTL cortex in “schema learning” (van Kesteren et al., 2012, 2013), this relationship was not significant in the parahippocampal cortex and was marginally dissociable from the relationship that we demonstrate in the hippocampal body. Prior research has demonstrated that hippocampal computations track distance to spatial locations (Sherrill et al., 2013; Howard et al., 2014; Chrastil et al., 2015; Spiers et al., 2018), and represent information about paths taken to get there (Wood et al., 2000; Ferbinteanu and Shapiro, 2003; Lee et al., 2006; Smith and Mizumori, 2006; Johnson and Redish, 2007; Ito et al., 2015; Wikenheiser and Redish, 2015; Brown et al., 2016). Our findings are consistent with this literature and, critically, indicate that prospective signals can emerge rapidly, after one-shot episodic learning.

In addition to demonstrating that prior spatial knowledge facilitates new learning, the present study explored whether prior knowledge facilitates the rapid emergence of prospective signals in the hippocampus that in turn supports navigational performance after one-shot learning. Our findings revealed a complex relationship between prior knowledge, the NPA encoding experience, and prospective hippocampal signals. Specifically, our estimate of trial-invariant OPA knowledge negatively related to hippocampal activity in a manner that was mediated by how quickly NPA locations were uncovered during block 1. By contrast, the most recent OPA experience (OPA block 12), while also qualitatively negatively related, did not significantly interact with hippocampal activity; this outcome converges with our exploratory follow-up analysis which showed that when both hippocampal activity and OPA_recent_ performance were entered as predictors in the model, they remained more robust independent predictors of subsequent NPA performance. These outcomes suggest that the relationship between prior knowledge and the rapid emergence of prospective signals after one-shot learning may be indirect. This process is potentially mediated not only by the complexity of the encoding experience, as indicated by our data, but also by processing in other circuitries in the brain.

One important area for future research will be to employ high-powered designs to explore the potential implications of the negative and null relationships between hippocampal activity and OPA knowledge metrics. While highly speculative, one hypothesis to explore is a facilitative relationship in which greater OPA (“schema”) knowledge could facilitate more *efficient* hippocampal retrieval—enabling recovery of more focal environmental information after just one learning trial (NPA block 1). In this model, the hippocampus would be engaged to facilitate retrieval relevant for navigational planning after one-shot learning, as observed here, but increased BOLD activity may be offset by a more precise memory facilitated by an existing spatial schema. Another speculative interpretation of our results is that they fall within a “schema consolidation” perspective of memory (Morris, 2006; Tse et al., 2007; van Kesteren et al., 2012; Gilboa and Marlatte, 2017)—when participants form more robust knowledge of the OPA location during Day 1 training (reflected by trial-invariant OPA performance on Day 2), new associations may be integrated into the spatial memory structure in a manner that decreases dependence on the hippocampus for retrieval (van Kesteren et al., 2012). Given recent interest in the interplay between the MTL and mPFC in updating of knowledge networks (van Kesteren et al., 2012; Gilboa and Marlatte, 2017), it is interesting to note that mPFC appears to be functionally coupled with the hippocampus during NPA retrieval after one-shot learning. By contrast, however, mPFC was not a significant predictor of subsequent NPA performance, nor was mPFC activity significantly reduced in relation to greater prior knowledge. Here, our present design did not allow us to directly address whether mPFC activity during initial encoding provides a mechanism for accelerated cortical learning that does not depend on the hippocampus (e.g., SLIMM—van Kesteren et al., 2012). However, it is possible that mPFC may modulate hippocampal predictive signals during early learning in the presence of prior knowledge, and this will be an interesting area for continued research.

Together, our results suggest that recent OPA retrieval experiences may govern effects of prior spatial knowledge on new spatial learning, or at least significantly mediate the relationship between trial-invariant spatial knowledge and new learning. Trial-invariant spatial knowledge was less directly related to NPA one-shot learning success but interacted with the behavioral experience during initial NPA encoding to mediate hippocampal involvement in prospective navigation after one-shot learning. Although a mechanistic account bridging our prior knowledge metrics and hippocampal prospective activity after one-shot learning will require further study to address, our results suggest that the hippocampus is involved in relating old to new spatial knowledge, just as in rodent studies (Tse et al., 2007; McKenzie et al., 2013, 2014; Richards et al., 2014) and some human studies including spatial (van Buuren et al., 2014; Sommer, 2017) and, less consistently, non-spatial learning (Liu et al., 2017). Our findings are generative, motivating further research into whether these hippocampal effects are specific to schema-enhanced learning in spatial and navigational settings, or whether they generalize to other non-spatial learning contexts (as might be expected by integrative encoding accounts of hippocampal memory function; Shohamy and Wagner, 2008).

Some limitations with our design should be mentioned. Because of scanning time constraints and concerns about subject fatigue, we could not include a non-schema condition where participants learned a location in new rooms, (i.e., rooms without prior knowledge). This would be an interesting addition for future research. Also, because we wanted to equate perceptual input over NPA trials as much as possible, and because we had only three learning opportunities for NPA, we decided to have participants start at the same position on every block. For this reason, NPA learning could have been more egocentric than OPA learning, where participants started from different corners and were allowed to develop a more allocentrically focused memory. This distinction would be another useful consideration for future research.

Here, we focused on brain activity during the block 2 planning period. This is because we could not interpret activity-behavior relationships in block 1 as a memory-based search (as targeted by our study). Participants typically employed a highly distinctive search strategy (e.g., a spiral) on the first block across rooms that had no clear relationship to OPA location. As such, behavior and activity in block 1 would be dominated by implementing an environment-independent search strategy rather than any trace memory for the NPA location (which they had not encountered yet). Moreover, we could not relate behavior in block 3 to one-shot learning, because there had already been two learning possibilities prior to this block.

Interestingly, prospective NPA performance relationships were significantly related to neural activity in the body of the hippocampus. Prior literature has emphasized the potential importance of the hippocampal tail for spatial memory and successful planning towards goals (Fanselow and Dong, 2010; Sherrill et al., 2013; Miller et al., 2017), motivating our analysis of hippocampal function based on rostro-caudal subdivisions. In our data, the tail was not the locus of the most significant relationship with NPA performance, although we note that the ability of the body to predict subsequent NPA performance independently from the tail only approached marginal significance (*p* = 0.11). Interestingly, however, activity in the hippocampal body was a significant independent predictor of NPA performance from the head, suggesting functional differentiation in the anterior extent of the hippocampus. Importantly, recent work suggests prospective goal coding is distributed along the long-axis of the human hippocampus (Brown et al., 2016), rather than localizing to the tail, and although our results were somewhat unexpected they underscore the importance of evaluating hippocampal function at the level of rostro-caudal subdivisions. In turn, although the dissociation between the hippocampal body and parahippocampal cortex was only marginally significant at traditional alpha thresholds, our data suggest the hippocampal body may also make a distinguishable contribution to prospective navigation after one-shot learning from the parahippocampal cortex, in which predictive effects did not approach significance. As with the dissociation between the body and the head, this result should be interpreted with caution, but it is nevertheless interesting given that parahippocampal cortex is well-established to support spatial scene processing and is often found to support navigational performance in other contexts (Epstein, 2008; Weniger et al., 2010; Howard et al., 2011; Brown et al., 2014, 2016; Marchette et al., 2015; Epstein et al., 2017). In particular, remote and well-practiced spatial knowledge may be more reliant on parahippocampal cortex than the hippocampus (Rosenbaum et al., 2004; Moscovitch et al., 2006). One influential theory of MTL memory function juxtaposes rapid memory formation in the hippocampus with more gradual learning mechanisms that could be supported by MTL cortex (McClelland et al., 1995; Kumaran and McClelland, 2012). Therefore, one possibility is that significant parahippocampal prospective memory signals may require more repetitions to emerge than our critical one-shot NPA learning measure indicative of schema learning benefits. Another possibility is that the parahippocampal cortex’s more general role in processing scene information (Epstein et al., 1999, 2017; Epstein, 2008; Marchette et al., 2015) contributes important information for performance on our task that is nevertheless less directly related to trial-wise subsequent navigation performance.

Lastly, when considering the relationship between precision of OPA knowledge and new learning, it was notable that the most recent measure (i.e., performance on OPA block 12) was more predictive of NPA learning than average performance on Day 2 retrieval blocks, which may offer a more pure representation of the level of consolidated knowledge (Dudai et al., 2015) following Day 1 training. This is an important finding, because the world is in constant flux, and the structure of our spatial memories may quantifiably evolve with continued practice in an environment (McKenzie et al., 2013). Our data suggest that the vagaries of recent retrieval-practice experience (Hulbert and Norman, 2015; Antony et al., 2017) with an individual environment influence how effectively new information about that environment is encoded and retrieved. Importantly, this OPA_recent_ measure is not independent from the overall level of knowledge of a given environment (indeed, it was correlated with OPA_average_, as one would expect). Consequently, OPA_recent_ may relate most-closely to one-shot NPA learning due to the fact that it carries information about both the overall level of knowledge about an environment going into Day 2 and participants’ current state of retrieval success, fatigue, interference between environment memories, and other factors that would be unique to the most recent trial in each environment. Therefore, our data suggest that there may be important insights to be uncovered when research into the effects of prior knowledge on new learning measures examines the influences of the most recent experience with accessing that prior knowledge. One potentially fruitful future direction will be to explore the relationship between the rapid NPA learning and retrieval effects demonstrated by our work and mechanisms of reconsolidation (Hupbach et al., 2007, 2008; Tronson and Taylor, 2007; Sederberg et al., 2011). NPA learning can be viewed as a re-experiencing of the learned environment in the context of a new navigational goal, and it may be possible to design variants of this task in which changes to a learned spatial memory engram from OPA to NPA learning experiences are quantifiable.

Alternative outcomes to what we observed might be predicted by other theories and empirical phenomena. In particular, the phenomenon of “blocking” (e.g., Hamilton and Sutherland, 1999) and, more broadly, the existence of proactive interference (e.g., Underwood, 1949) might lead to the prediction that environments in which the OPA was better learned would be the ones in which it would be harder to learn the NPA. Again, our findings demonstrated the opposite, as better OPA learning predicts better NPA learning, consistent with the schema-enhanced learning hypothesis (along with other evidence of the benefits of mnemonic integration; e.g., Shohamy and Wagner, 2008; Kuhl et al., 2010).

In conclusion, our findings revealed a relationship between the precision of prior spatial knowledge and new spatial learning in humans. Moreover, the hippocampus prospectively codes the precision of this new spatial learning after one-shot learning. Finally, prior knowledge and the complexity of encoding experiences appear to interact with prospective hippocampal signals that support one-shot learning behavior. These relationships could arise through several mechanisms, and thus our findings help frame future research in this area. The present results extend the current human schema literature, offering important insights into the behavioral manifestations of spatial knowledge that can give rise to enhanced new learning, and suggesting a potential role for the hippocampus in translating one-shot spatial learning that is facilitated by prior knowledge into prospective navigational planning.

## Author Contributions

MvK, TB and AW designed the experiment and wrote the article. MvK collected the data. MvK and TB analyzed the data.

## Conflict of Interest Statement

The authors declare that the research was conducted in the absence of any commercial or financial relationships that could be construed as a potential conflict of interest.

## Footnotes

^1^http://psychtoolbox.org

^2^http://www.fil.ion.ucl.ac.uk/spm

^3^http://www.nitrc.org/projects/artifact_detect/

^4^http://www.itksnap.org

## References

[B1] Addis D. R. Wong A. T. Schacter D. L. (2007). Remembering the past and imagining the future: common and distinct neural substrates during event construction and elaboration. Neuropsychologia 45, 1363–1377. 10.1016/j.neuropsychologia.2006.10.016 17126370 PMC1894691

[B2] Alt H. Godau M. (1995). Computing the frechet distance between 2 polygonal curves. Int. J. Comput. Geom. Appl. 5, 75–91. 10.1142/s0218195995000064

[B3] Antony J. W. Ferreira C. S. Norman K. A. Wimber M. (2017). Retrieval as a fast route to memory consolidation. Trends Cogn. Sci. 21, 573–576. 10.1016/j.tics.2017.05.001 28583416 PMC5912918

[B4] Ashburner J. (2007). A fast diffeomorphic image registration algorithm. Neuroimage 38, 95–113. 10.1016/j.neuroimage.2007.07.007 17761438

[B5] Barr D. J. Levy R. Scheepers C. Tily H. J. (2013). Random effects structure for confirmatory hypothesis testing: keep it maximal. J. Mem. Lang. 68, 255–278. 10.1016/j.jml.2012.11.001 24403724 PMC3881361

[B6] Bartlett F. C. (1932). Remembering: A Study in Experimental and Social Psychology. Cambridge, MA: University Press.

[B7] Brod G. Lindenberger U. Werkle-Bergner M. Shing Y. L. (2015). Differences in the neural signature of remembering schema-congruent and schema-incongruent events. Neuroimage 117, 358–366. 10.1016/j.neuroimage.2015.05.086 26048620

[B9] Brown T. I. Carr V. A. LaRocque K. F. Favila S. E. Gordon A. M. Bowles B.. (2016). Prospective representation of navigational goals in the human hippocampus. Science 352, 1323–1326. 10.1126/science.aaf0784 27284194

[B10] Brown T. I. Hasselmo M. E. Stern C. E. (2014). A high-resolution study of hippocampal and medial temporal lobe correlates of spatial context and prospective overlapping route memory. Hippocampus 24, 819–839. 10.1002/hipo.22273 24659134 PMC4455903

[B8] Brown T. I. Stern C. E. (2014). Contributions of medial temporal lobe and striatal memory systems to learning and retrieving overlapping spatial memories. Cereb. Cortex 24, 1906–1922. 10.1093/cercor/bht041 23448868 PMC4051896

[B11] Buckner R. L. Carroll D. C. (2007). Self-projection and the brain. Trends Cogn. Sci. 11, 49–57. 10.1016/j.tics.2006.11.004 17188554

[B12] Burgess N. Maguire E. A. O’keefe J. (2002). The human hippocampus and spatial and episodic memory. Neuron 35, 625–641. 10.1016/s0896-6273(02)00830-9 12194864

[B13] Buzsáki G. (2005). Theta rhythm of navigation: link between path integration and landmark navigation, episodic and semantic memory. Hippocampus 15, 827–840. 10.1002/hipo.20113 16149082

[B14] Buzsáki G. Moser E. I. (2013). Memory, navigation and theta rhythm in the hippocampal-entorhinal system. Nat. Neurosci. 16, 130–138. 10.1038/nn.3304 23354386 PMC4079500

[B15] Chrastil E. R. Sherrill K. R. Hasselmo M. E. Stern C. E. (2015). There and back again: hippocampus and retrosplenial cortex track homing distance during human path integration. J. Neurosci. 35, 15442–15452. 10.1523/JNEUROSCI.1209-15.2015 26586830 PMC6605486

[B16] Doeller C. F. King J. A. Burgess N. (2008). Parallel striatal and hippocampal systems for landmarks and boundaries in spatial memory. Proc. Natl. Acad. Sci. U S A 105, 5915–5920. 10.1073/pnas.0801489105 18408152 PMC2311337

[B17] Dudai Y. Karni A. Born J. (2015). The consolidation and transformation of memory. Neuron 88, 20–32. 10.1016/j.neuron.2015.09.004 26447570

[B18] Duvernoy H. M. (2005). The Human Hippocampus: Functional Anatomy, Vascularization and Serial Sections With MRI. Berlin, Heidelberg: Springer-Verlag.

[B19] Eichenbaum H. Yonelinas A. P. Ranganath C. (2007). The medial temporal lobe and recognition memory. Annu. Rev. Neurosci. 30, 123–152. 10.1146/annurev.neuro.30.051606.094328 17417939 PMC2064941

[B20] Ekstrom A. D. Bookheimer S. Y. (2007). Spatial and temporal episodic memory retrieval recruit dissociable functional networks in the human brain. Learn. Mem. 14, 645–654. 10.1101/lm.575107 17893237 PMC2044556

[B21] Ekstrom A. D. Arnold A. E. G. F. Iaria G. (2014). A critical review of the allocentric spatial representation and its neural underpinnings: toward a network-based perspective. Front. Hum. Neurosci. 8:803. 10.3389/fnhum.2014.00803 25346679 PMC4193251

[B23] Epstein R. A. (2008). Parahippocampal and retrosplenial contributions to human spatial navigation. Trends Cogn. Sci. 12, 388–396. 10.1016/j.tics.2008.07.004 18760955 PMC2858632

[B22] Epstein R. Harris A. Stanley D. Kanwisher N. (1999). The parahippocampal place area: recognition, navigation, or encoding? Neuron 23, 115–125. 10.1016/s0896-6273(00)80758-8 10402198

[B24] Epstein R. A. Patai E. Z. Julian J. B. Spiers H. J. (2017). The cognitive map in humans: spatial navigation and beyond. Nat. Neurosci. 20, 1504–1513. 10.1038/nn.4656 29073650 PMC6028313

[B25] Fanselow M. S. Dong H. W. (2010). Are the dorsal and ventral hippocampus functionally distinct structures? Neuron 65, 7–19. 10.1016/j.neuron.2009.11.031 20152109 PMC2822727

[B26] Ferbinteanu J. Shapiro M. L. (2003). Prospective and retrospective memory coding in the hippocampus. Neuron 40, 1227–1239. 10.1016/s0896-6273(03)00752-9 14687555

[B27] Gilboa A. Marlatte H. (2017). Neurobiology of schemas and schema-mediated memory. Trends Cogn. Sci. 21, 618–631. 10.1016/j.tics.2017.04.013 28551107

[B28] Hamilton D. A. Sutherland R. J. (1999). Blocking in human place learning: evidence from virtual navigation. Psychobiology 27, 453–461.

[B29] Hartley T. Maguire E. A. Spiers H. J. Burgess N. (2003). The well-worn route and the path less traveled: distinct neural bases of route following and wayfinding in humans. Neuron 37, 877–888. 10.1016/S0896-6273(03)00095-3 12628177

[B30] Howard L. R. Javadi A. H. Yu Y. Mill R. D. Morrison L. C. Knight R.. (2014). The hippocampus and entorhinal cortex encode the path and Euclidean distances to goals during navigation. Curr. Biol. 24, 1331–1340. 10.1016/j.cub.2014.05.001 24909328 PMC4062938

[B31] Howard L. R. Kumaran D. Ólafsdóttir H. F. Spiers H. J. (2011). Double dissociation between hippocampal and parahippocampal responses to object-background context and scene novelty. J. Neurosci. 31, 5253–5261. 10.1523/JNEUROSCI.6055-10.2011 21471360 PMC3079899

[B32] Hulbert J. C. Norman K. A. (2015). Neural differentiation tracks improved recall of competing memories following interleaved study and retrieval practice. Cereb. Cortex 25, 3994–4008. 10.1093/cercor/bhu284 25477369 PMC4585527

[B33] Hupbach A. Gomez R. Hardt O. Nadel L. (2007). Reconsolidation of episodic memories: a subtle reminder triggers integration of new information. Learn. Mem. 14, 47–53. 10.1101/lm.365707 17202429 PMC1838545

[B34] Hupbach A. Hardt O. Gomez R. Nadel L. (2008). The dynamics of memory: context-dependent updating. Learn. Mem. 15, 574–579. 10.1101/lm.1022308 18685148

[B35] Iaria G. Petrides M. Dagher A. Pike B. Bohbot V. D. (2003). Cognitive strategies dependent on the hippocampus and caudate nucleus in human navigation: variability and change with practice. J. Neurosci. 23, 5945–5952. 10.1523/JNEUROSCI.23-13-05945.2003 12843299 PMC6741255

[B36] Insausti R. Juottonen K. Soininen H. Insausti A. M. Partanen K. Vainio P.. (1998). MR volumetric analysis of the human entorhinal, perirhinal, and temporopolar cortices. Am. J. Neuroradiol. 19, 659–671. 9576651 PMC8337393

[B37] Ito H. T. Zhang S. J. Witter M. P. Moser E. I. Moser M. B. (2015). A prefrontal-thalamo-hippocampal circuit for goal-directed spatial navigation. Nature 522, 50–55. 10.1038/nature14396 26017312

[B38] Johnson A. Redish A. D. (2007). Neural ensembles in CA3 transiently encode paths forward of the animal at a decision point. J. Neurosci. 27, 12176–12189. 10.1523/JNEUROSCI.3761-07.2007 17989284 PMC6673267

[B39] Kuhl B. A. Shah A. T. DuBrow S. Wagner A. D. (2010). Resistance to forgetting associated with hippocampus-mediated reactivation during new learning. Nat. Neurosci. 13, 501–506. 10.1038/nn.2498 20190745 PMC2847013

[B40] Kumaran D. McClelland J. L. (2012). Generalization through the recurrent interaction of episodic memories: a model of the hippocampal system. Psychol. Rev. 119, 573–616. 10.1037/a0028681 22775499 PMC3444305

[B41] Lee I. Griffin A. L. Zilli E. A. Eichenbaum H. Hasselmo M. E. (2006). Gradual translocation of spatial correlates of neuronal firing in the hippocampus toward prospective reward locations. Neuron 51, 639–650. 10.1016/j.neuron.2006.06.033 16950161

[B42] Lee J. L. C. Nader K. Schiller D. (2017). An update on memory reconsolidation updating. Trends Cogn. Sci. 21, 531–545. 10.1016/j.tics.2017.04.006 28495311 PMC5605913

[B43] Liu Z. X. Grady C. Moscovitch M. (2017). Effects of prior-knowledge on brain activation and connectivity during associative memory encoding. Cereb. Cortex 27, 1991–2009. 10.1093/cercor/bhw047 26941384

[B44] Marchette S. A. Vass L. K. Ryan J. Epstein R. A. (2015). Outside looking in: landmark generalization in the human navigational system. J. Neurosci. 35, 14896–14908. 10.1523/JNEUROSCI.2270-15.2015 26538658 PMC4635136

[B45] McClelland J. L. McNaughton B. L. O’Reilly R. C. (1995). Why there are complementary learning systems in the hippocampus and neocortex: insights from the successes and failures of connectionist models of learning and memory. Psychol. Rev. 102, 419–457. 10.1037/0033-295x.102.3.419 7624455

[B46] McKenzie S. Frank A. J. Kinsky N. R. Porter B. Rivière P. D. Eichenbaum H. (2014). Hippocampal representation of related and opposing memories develop within distinct, hierarchically organized neural schemas. Neuron 83, 202–215. 10.1016/j.neuron.2014.05.019 24910078 PMC4082468

[B47] McKenzie S. Robinson N. T. Herrera L. Churchill J. C. Eichenbaum H. (2013). Learning causes reorganization of neuronal firing patterns to represent related experiences within a hippocampal schema. J. Neurosci. 33, 10243–10256. 10.1523/JNEUROSCI.0879-13.2013 23785140 PMC3685831

[B48] McNamara T. P. Shelton A. L. Shelton A. L. (2003). Cognitive maps and the hippocampus. Trends Cogn. Sci. 7, 333–335. 10.1016/s1364-6613(03)00167-0 12907223

[B49] McVee M. B. Dunsmore K. Gavelek J. R. (2005). Schema theory revisited. Rev. Educ. Res. 75, 531–566. 10.3102/00346543075004531

[B50] Miller K. J. Botvinick M. M. Brody C. D. (2017). Dorsal hippocampus contributes to model-based planning. Nat. Neurosci. 20, 1269–1276. 10.1038/nn.4613 28758995 PMC5575950

[B51] Morris R. G. (2006). Elements of a neurobiological theory of hippocampal function: the role of synaptic plasticity, synaptic tagging and schemas. Eur. J. Neurosci. 23, 2829–2846. 10.1111/j.1460-9568.2006.04888.x 16819972

[B52] Moscovitch M. Nadel L. Winocur G. Gilboa A. Rosenbaum R. S. (2006). The cognitive neuroscience of remote episodic, semantic and spatial memory. Curr. Opin. Neurobiol. 16, 179–190. 10.1016/j.conb.2006.03.013 16564688

[B53] Pazzaglia F. De Beni R. (2001). Strategies of processing spatial information in survey and landmark-centered individuals. Eur. J. Cogn. Psychol. 13, 493–508. 10.1080/09541440042000124

[B54] Poppenk J. Evensmoen H. R. Moscovitch M. Nadel L. (2013). Long-axis specialization of the human hippocampus. Trends Cogn. Sci. 17, 230–240. 10.1016/j.tics.2013.03.005 23597720

[B55] Preston A. R. Eichenbaum H. (2013). Interplay of hippocampus and prefrontal cortex in memory. Curr. Biol. 23, R764–R773. 10.1016/j.cub.2013.05.041 24028960 PMC3789138

[B56] Preston A. R. Bornstein A. M. Hutchinson J. B. Gaare M. E. Glover G. H. Wagner A. D. (2010). High-resolution fMRI of content-sensitive subsequent memory responses in human medial temporal lobe. J. Cogn. Neurosci. 22, 156–173. 10.1162/jocn.2009.21195 19199423 PMC2854293

[B57] Pruessner J. C. Köhler S. Crane J. Pruessner M. Lord C. Byrne A.. (2002). Volumetry of temporopolar, perirhinal, entorhinal and parahippocampal cortex from high-resolution MR images: considering the variability of the collateral sulcus. Cereb. Cortex 12, 1342–1353. 10.1093/cercor/12.12.1342 12427684

[B58] Pruessner J. C. Li L. M. Serles W. Pruessner M. Collins D. L. Kabani N.. (2000). Volumetry of hippocampus and amygdala with high-resolution MRI and three-dimensional analysis software: minimizing the discrepancies between laboratories. Cereb. Cortex 10, 433–442. 10.1093/cercor/10.4.433 10769253

[B59] R Core Team (2012). R: A Language and Environment for Statistical Computing. Vienna: R Foundation for Statistical Computing.

[B60] Ranganath C. Ritchey M. (2012). Two cortical systems for memory-guided behavior. Nat. Rev. Neurosci. 13, 713–726. 10.1038/nrn3338 22992647

[B61] Richards B. A. Xia F. Santoro A. Husse J. Woodin M. A. Josselyn S. A.. (2014). Patterns across multiple memories are identified over time. Nat. Neurosci. 17, 981–986. 10.1038/nn.3736 24880213

[B62] Rosenbaum R. S. Ziegler M. Winocur G. Grady C. L. Moscovitch M. (2004). “I have often walked down this street before”: fMRI studies on the hippocampus and other structures during mental navigation of an old environment. Hippocampus 14, 826–835. 10.1002/hipo.10218 15382253

[B63] Schacter D. L. Addis D. R. (2009). On the nature of medial temporal lobe contributions to the constructive simulation of future events. Philos. Trans. R. Soc. Lond. B Biol. Sci. 364, 1245–1253. 10.1098/rstb.2008.0308 19528005 PMC2666708

[B64] Schlichting M. L. Preston A. R. (2015). Memory integration: neural mechanisms and implications for behavior. Curr. Opin. Behav. Sci. 1, 1–8. 10.1016/j.cobeha.2014.07.005 25750931 PMC4346341

[B65] Sederberg P. B. Gershman S. J. Polyn S. M. Norman K. A. (2011). Human memory reconsolidation can be explained using the temporal context model. Psychon. Bull. Rev. 18, 455–468. 10.3758/s13423-011-0086-9 21512839 PMC3432313

[B66] Sherrill K. R. Erdem U. M. Ross R. S. Brown T. I. Hasselmo M. E. Stern C. E. (2013). Hippocampus and retrosplenial cortex combine path integration signals for successful navigation. J. Neurosci. 33, 19304–19313. 10.1523/JNEUROSCI.1825-13.2013 24305826 PMC3850045

[B67] Shohamy D. Wagner A. D. (2008). Integrating memories in the human brain: hippocampal-midbrain encoding of overlapping events. Neuron 60, 378–389. 10.1016/j.neuron.2008.09.023 18957228 PMC2628634

[B68] Smith D. M. Mizumori S. J. (2006). Learning-related development of context-specific neuronal responses to places and events: the hippocampal role in context processing. J. Neurosci. 26, 3154–3163. 10.1523/JNEUROSCI.3234-05.2006 16554466 PMC6674095

[B69] Sommer T. (2017). The emergence of knowledge and how it supports the memory for novel related Information. Cereb. Cortex 27, 1906–1921. 10.1093/cercor/bhw031 26908636

[B70] Spiers H. J. Olafsdottir H. F. Lever C. (2018). Hippocampal CA1 activity correlated with the distance to the goal and navigation performance. Hippocampus 28, 644–658. 10.1002/hipo.22813 29149774 PMC6282985

[B71] Squire L. R. Stark C. E. Clark R. E. (2004). The medial temporal lobe. Annu. Rev. Neurosci. 27, 279–306. 10.1146/annurev.neuro.27.070203.144130 15217334

[B72] Stefanacci L. Buffalo E. A. Schmolck H. Squire L. R. (2000). Profound amnesia after damage to the medial temporal lobe: a neuroanatomical and neuropsychological profile of patient E. P. J. Neurosci. 20, 7024–7036. 10.1523/JNEUROSCI.20-18-07024.2000 10995848 PMC6772843

[B73] Tronson N. C. Taylor J. R. (2007). Molecular mechanisms of memory reconsolidation. Nat. Rev. Neurosci. 8, 262–275. 10.1038/nrn2090 17342174

[B74] Tse D. Langston R. F. Kakeyama M. Bethus I. Spooner P. A. Wood E. R.. (2007). Schemas and memory consolidation. Science 316, 76–82. 10.1126/science.1135935 17412951

[B75] Tse D. Takeuchi T. Kakeyama M. Kajii Y. Okuno H. Tohyama C.. (2011). Schema-dependent gene activation and memory encoding in neocortex. Science 333, 891–895. 10.1126/science.1205274 21737703

[B76] Underwood B. J. (1949). Proactive inhibition as a function of time and degree of prior learning. J. Exp. Psychol. 39, 24–34. 10.1037/h0059550 18111558

[B77] van Buuren M. Kroes M. C. Wagner I. C. Genzel L. Morris R. G. Fernández G. (2014). Initial investigation of the effects of an experimentally learned schema on spatial associative memory in humans. J. Neurosci. 34, 16662–16670. 10.1523/JNEUROSCI.2365-14.2014 25505319 PMC6608500

[B78] van Kesteren M. T. Beul S. F. Takashima A. Henson R. N. Ruiter D. J. Fernández G. (2013). Differential roles for medial prefrontal and medial temporal cortices in schema-dependent encoding: from congruent to incongruent. Neuropsychologia 51, 2352–2359. 10.1016/j.neuropsychologia.2013.05.027 23770537

[B79] van Kesteren M. T. Brown T. I. Wagner A. D. (2016). Interactions between memory and new learning: insights from fMRI multivoxel pattern analysis. Front. Syst. Neurosci. 10:46. 10.3389/fnsys.2016.00046 27303274 PMC4880566

[B80] van Kesteren M. T. Fernández G. Norris D. G. Hermans E. J. (2010a). Persistent schema-dependent hippocampal-neocortical connectivity during memory encoding and postencoding rest in humans. Proc. Natl. Acad. Sci. U S A 107, 7550–7555. 10.1073/pnas.0914892107 20363957 PMC2867741

[B81] van Kesteren M. T. Rijpkema M. Ruiter D. J. Fernández G. (2010b). Retrieval of associative information congruent with prior knowledge is related to increased medial prefrontal activity and connectivity. J. Neurosci. 30, 15888–15894. 10.1523/JNEUROSCI.2674-10.2010 21106827 PMC6633736

[B82] van Kesteren M. T. Ruiter D. J. Fernandez G. Henson R. N. (2012). How schema and novelty augment memory formation. Trends Neurosci. 35, 211–219. 10.1016/j.tins.2012.02.001 22398180

[B83] Voermans N. C. Petersson K. M. Daudey L. Weber B. Van Spaendonck K. P. Kremer H. P.. (2004). Interaction between the human hippocampus and the caudate nucleus during route recognition. Neuron 43, 427–435. 10.1016/j.neuron.2004.07.009 15294149

[B84] Wagner I. C. van Buuren M. Kroes M. C. Gutteling T. P. van der Linden M. Morris R. G.. (2015). Schematic memory components converge within angular gyrus during retrieval. Elife 4:e09668. 10.7554/elife.09668 26575291 PMC4709269

[B85] Weniger G. Irle E. (2006). Posterior parahippocampal gyrus lesions in the human impair egocentric learning in a virtual environment. Eur. J. Neurosci. 24, 2406–2414. 10.1111/j.1460-9568.2006.05108.x 17074058

[B86] Weniger G. Siemerkus J. Schmidt-Samoa C. Mehlitz M. Baudewig J. Dechent P.. (2010). The human parahippocampal cortex subserves egocentric spatial learning during navigation in a virtual maze. Neurobiol. Learn. Mem. 93, 46–55. 10.1016/j.nlm.2009.08.003 19683063

[B87] Wikenheiser A. M. Redish A. D. (2015). Hippocampal theta sequences reflect current goals. Nat. Neurosci. 18, 289–294. 10.1038/nn.3909 25559082 PMC4428659

[B88] Wolbers T. Büchel C. (2005). Dissociable retrosplenial and hippocampal contributions to successful formation of survey representations. J. Neurosci. 25, 3333–3340. 10.1523/JNEUROSCI.4705-04.2005 15800188 PMC6724902

[B89] Wolbers T. Wiener J. M. (2014). Challenges for identifying the neural mechanisms that support spatial navigation: the impact of spatial scale. Front. Hum. Neurosci. 8:571. 10.3389/fnhum.2014.00571 25140139 PMC4121531

[B90] Wood E. R. Dudchenko P. A. Robitsek R. J. Eichenbaum H. (2000). Hippocampal neurons encode information about different types of memory episodes occurring in the same location. Neuron 27, 623–633. 10.1016/s0896-6273(00)00071-4 11055443

[B91] Yassa M. A. Stark C. E. (2009). A quantitative evaluation of cross-participant registration techniques for MRI studies of the medial temporal lobe. Neuroimage 44, 319–327. 10.1016/j.neuroimage.2008.09.016 18929669

[B92] Yeo B. Krienen F. Sepulcre J. Sabuncu M. Lashkari D. Hollinshead M.. (2011). The organization of the human cerebral cortex estimated by intrinsic functional connectivity. J. Neurophysiol. 106, 1125–1165. 10.1152/jn.00338.2011 21653723 PMC3174820

[B93] Yushkevich P. A. Piven J. Hazlett H. C. Smith R. G. Ho S. Gee J. C.. (2006). User-guided 3D active contour segmentation of anatomical structures: significantly improved efficiency and reliability. Neuroimage 31, 1116–1128. 10.1016/j.neuroimage.2006.01.015 16545965

