# Supplementary material for: Learned Spatial Schemas and Prospective Hippocampal Activity Support Navigation After One-Shot Learning
Source: Front Hum Neurosci. 2018 Dec 4;12:486. doi: 10.3389/fnhum.2018.00486 (PMC6288548; doi:10.3389/fnhum.2018.00486)
Supplement: Supplementary file 1 [file Table_1.DOCX]

**Supplementary Information**

**Table S1**

| **Memory test (units)** | **Mean (SD) Males (n=8)** | **Mean (SD) Females (n=8)** | **T-value** | **P-value** |
| --- | --- | --- | --- | --- |
| OPA 1 (PE) | 1.16 (.22) | 1.43 (.40) | -.16 | .13 |
| OPA 2 (PE) | 3.35 (.55) | 3.70 (1.42) | -.65 | .53 |
| OPA 3 (PE) | 2.08 (.68) | 2.42 (1.00) | -.80 | .44 |
| OPA 4 (PE) | 1.78 (.40) | 1.78 (.81) | 0 | 1 |
| AT 1 (%) | 49.75 (27.91) | 57.75 (25.49) | -.60 | .56 |
| OPA 5 (PE) | 1.44 (.28) | 1.61 (.43) | -.95 | .36 |
| OPA 6 (PE) | 1.34 (.30) | 1.37 (.30) | -.20 | .85 |
| OPA 7 (PE) | 1.33 (.25) | 1.23 (.16) | .98 | .34 |
| OPA 8 (PE) | 1.31 (.32) | 1.29 (.22) | .14 | .89 |
| AT 2 (%) | 87.88 (16.95) | 91.25 (14.99) | -.42 | .68 |
| OPA 9 (PE) | 1.24 (.18) | 1.26 (.26) | -.21 | .84 |
| OPA 10 (PE) | 1.24 (.17) | 1.27 (.20) | .25 | .81 |
| OPA 11 (PE) | 1.23 (.16) | 1.22 (.16) | .10 | .92 |
| OPA 12 (PE) | 1.14 (.07) | 1.20 (.18) | -.88 | .40 |
| AT 3 (%) | 96.50 (5.32) | 98.50 (2.27) | -.98 | .35 |
| NPA 1 (PE) | 4.52 (1.42) | 5.11 (1.70) | -.75 | .47 |
| NPA 2 (PE) | 2.59 (.79) | 2.25 (.57) | .98 | .34 |
| NPA 3 (PE) | 1.71 (.61) | 1.70 (.60) | .04 | .97 |
| Post-scan NPA wall (ED) | 7.51 (2.45) | 7.44 (2.47) | .06 | .96 |
| Post-scan OPA (ED) | 6.22 (1.61) | 6.41 (2.07) | -.21 | .84 |
| Post-scan OPA-NPA (ED) | 8.54 (3.25) | 8.55 (3.53) | 0 | 1 |

*Table S1. Gender differences. Exploratory analyses examining gender differences on the memory tests (in order of time). Analyses were performed using an Independent Samples T-test. None of these comparisons proved significant. NB: please note the low participant number (n=8) in both groups. (OPA = Old Paired Associate, NPA = New Paired Associate, AT = Associative Test, PE = Path Efficiency, NPA wall = NPA distance cued by wall, OPA-NPA = OPA distance cued by OPA, ED = Euclidean distance)*
